# Role of epinephrine in attenuating cytokine storm, decreasing ferritin, and inhibiting ferroptosis in SARS-CoV-2

**DOI:** 10.1186/s43044-024-00455-9

**Published:** 2024-02-20

**Authors:** Ashraf EL-Molla, Fawzia Aboul Fetouh, Samir Bawazir, Yasser Ali, Yehya Alwahby, Muhammad Bahadeg, Yousef Gotah, Fatima Ahmed Badahdah, Abdullatif H. Alsaeed, Abdullah Basseet

**Affiliations:** 1 ALKAWKAB Hospital, Cairo, Egypt; 2 Misr University for Science and Technology, Cairo, Egypt; 3 Department of Otorhinolaryngology, Head and Neck Surgery, Prince Sultan Military Medical City, Riyadh, Kingdom of Saudi Arabia; 4 Prince Sultan Military Medical City, Riyadh, Kingdom of Saudi Arabia; 5 King Fahd Medical City, Riyadh, Kingdom of Saudi Arabia; 6 King Faisal Specialist Hospital and Research Center (KFSHRC), Jeddah, Kingdom of Saudi Arabia

**Keywords:** SARS-CoV-2, Epinephrine, Ferritin, Ferroptosis, Cytokine storm

## Abstract

**Background:**

Severe acute respiratory syndrome coronavirus 2 (SARS-CoV-2) is the virus responsible for coronavirus disease 2019. It presents one of the most threatening pandemics in the history of humanity. The mortality and morbidity represent an unprecedented challenge to the modern medical era. SARS-CoV-2 results in acute respiratory distress syndrome, high concentrations of proinflammatory mediators, cytokine storm (CS) due to massive release of cytokines, hypercoagulation, and hemoglobin disintegration. Dysregulation of iron homeostasis, iron overload as indicated by high ferritin level, and ferroptosis are major factors in the pathogenesis of the disease. We report a case of SARS-CoV-2 in which the use of epinephrine (Epi) resulted in an unexpected attenuation of CS, decreasing ferritin level and inhibiting ferroptosis.

**Case presentation:**

A 64-year-old male patient with a history of multiple medical comorbidities had been diagnosed with SARS-CoV-2. Further evaluation showed marked increase in inflammatory markers, severe hyperferritinemia, and lymphopenia in laboratory blood tests. The characteristic score of CS was strongly positive, and in addition to regular treatment, the patient received Epi due to development of acute generalized skin rash, severe itching, and edema of lips and tongue. Epi may have successfully terminated not only the acute cutaneous condition, but also have attenuated CS, decreased ferritin level, and other inflammatory markers in addition to complete patient’s recovery.

**Conclusion:**

Epinephrine may attenuate CS and inhibit ferroptosis which is an iron-dependent, non-apoptotic mode of cell death. Epi interacts with ferric and/or ferrous iron and built a stable complex that impedes activation of beta-adrenergic receptors. Epi may cause marked decrease of ferritin and other inflammatory markers. Epi may be used to decrease iron overload which is associated with many medical diseases like type 2 diabetes mellitus and cardiometabolic diseases such as coronary heart disease and cerebrovascular disease. As a new clinical indication extensive studies are required for further assessment and possible therapeutic uses.

## Background

SARS-CoV-2 is the virus responsible for coronavirus disease 2019 (COVID-19) which presents one of the most threatening pandemics in the history of humanity. The mortality and morbidity represent an unprecedented challenge to the modern medical era [1]. Until March 2023, nearly 761.07 million cases and 6.87 million deaths have been reported according to statistics from the World Health Organization [2]. Clinical manifestations are absent or mild in a substantial proportion of patients who test positive for SARS-CoV-2. However, bilateral pneumonia is the main finding in hospitalized patients [3] and at least 5% initially present in serious condition, and require admission to intensive care unit (ICU) [4]. SARS-CoV-2 infects cells by attaching [5] to angiotensin converting enzyme receptor 2. SARS-CoV-2 has many complications which include acute respiratory distress syndrome (ARDS) [6], high concentrations of proinflammatory mediators [7], CS due to massive release of cytokines [8], hypercoagulation [9], and hemoglobin disintegration [10]. Dysregulation of iron homeostasis [11], iron overload [12, 13], and ferroptosis are major factor in the pathogenesis of SARS-CoV-2 [14, 15]. Atypical presentation of ARDS is not only caused by alveolar damage [16, 17] but also due to vascular endothelial injury, destruction of the beta-1 chain of hemoglobin that releases iron into the circulation [10]. Increased iron overload is associated with increased blood viscosity as well as recurrent and diffuse micro and macro vascular thrombosis which leads to elevated levels of D-dimer and death in many cases [10, 16, 17]. In 2012, Dixon proposed the concept of ferroptosis, an iron-dependent, non-apoptotic mode of cell death characterized by ferritin degradation, lipid peroxidation, and accumulation of reactive oxygen species (ROS) [18]. Free unbounded iron (Fe^+2^) is characterized by a high reactivity and toxicity due to formation of free radical oxygen species (ROS) through Fenton and Haber–Weiss reaction [19]. ROS formation may contribute not only to lung injury, but also to increased endothelial permeability, increased cytokine level in the lungs, and neutrophilic alveolar infiltrates [20]. High serum ferritin is also associated with ARDS progression [20, 21], and as tissue damage progresses and iron level increases, ferritin increases also to isolate iron [21]. We report a case of SARS-CoV-2 in which Epi was used and resulted in an unexpected attenuation of CS, decreasing ferritin level and inhibiting ferroptosis. The mechanism of action and the new indication of Epi will be discussed.

## Case presentation

A 64-year-old male patient with a medical history of ischemic heart disease, previous coronary artery bypass grafting surgery, dyslipidemia, and cerebral transient ischemic attacks. He was receiving clopidogrel, atorvastatin, and bisoprolol. He presented with fever, dry cough, severe myalgia, and bony aches. Real-time reverse transcription-polymerase chain reaction assay of nasopharyngeal (NP) swab was positive for SARS-CoV-2. Clinical examination revealed fever: 38.8 °C (degree Celsius), blood pressure: 117/71 mmHg, heart rate: 113 beat/min, respiratory rate: 14  times/min, and peripheral oxygen saturation: 95% on room air. Chest examination revealed normal breath sounds in upper lung zones, while coarse crackles and wheezes were more in lower lung zones. Normal first and second heart sounds were detected and accompanied by mild tachycardia. Chest radiography showed bilateral mainly peripheral rounded opacities mostly in lower lung fields, and chest computed tomography revealed bilateral ground glass opacities with peripheral distribution mainly in lower lung fields. Serial laboratory blood tests are shown in Table 1. The patient was isolated and given acetaminophen 500 mg/6-h, azithromycin 500 mg/day for 10 days, rivaroxaban 10 mg/day for 3 weeks, clopidogrel 75 mg/day, prednisone 20 mg/day for 10 days followed by slow tapering every 3 days for 2 weeks, ascorbic acid 1 g/day, and zinc 30 mg/day. On the morning of the ninth day the peak level of serum ferritin and other inflammatory markers such as fibrinogen, C-reactive protein (CRP), D-dimer, lactate dehydrogenase (LDH), and lowest level of lymphocytes were found in the blood sample. On the night of the same day, the patient complained of generalized skin rash accompanied by severe itching. He received intravenous hydrocortisone and after 15 min the itching worsened as he developed swelling of lips and tongue. For fear of compromising his airway Epi 0.6 mcg/kg had been prescribed; thus, 50 mcg was injected subcutaneous (S/C) in the upper thigh. The dose was repeated after 20 min and resulted in successful resolution of itching, lips and tongue edema. Four hours later the same condition was recurred and resolved by injecting 50 mcg Epi S/C; meanwhile, the absence of tachycardia and hypertension was noticed. The cutaneous eruption continued for 5 days, and Epi was injected 4–5 times daily to manage this condition under medical supervision and monitoring. Serial biochemical laboratory tests showed normal renal and hepatic function tests and marked decrease of serum ferritin from 2000 to 855 ng/ml. Meanwhile, other inflammatory markers decreased markedly (Table 1), and the patient’s general condition improved dramatically, so he was discharged on day 20 after 2 negative NP swabs. Phlebotomy was done to avoid deleterious effects of elevated ferritin, and resulted in decreasing ferritin from 860 to 843 ng/ml. However, Epi was administered three times daily under monitoring and medical supervision and we observed a decrease of ferritin level to 258 ng/ ml in 3 weeks.

**Table 1:** Laboratory blood results

Date of illness	25 December	28 December	28 December	28 December	3 January	5 January	7 January	9 January	12 January	14 January	19 January	24 January	29 January	3 February
Day number	Day(D) 1	D3	D5	D7	D9	D11	D13	D15	D18	D20	D25	D30	D35	D40
Lymphocytes	3.46	2.85	2.1	1.1	0.92	0.98	1.11	1.52	1.91	2.22	2.11	2.41	3.14	3.21
Ferritin	461	701	950	1600	2000	1410	1150	855	860	843	755	629	503	258
D-dimer	0.53	0.78	0.91	1.21	1.48	1.25	1.11	0.54	0.51	0.48	0.42	0.39	0.41	0.32
CRP	13.6	74.3	89.1	110.1	164.8	91.1	65.1	9.2	5.9	4.9	3.9	3.0	1.0	0.8
LDH	240	285	301	310	317	299	241	231	210	221	201	185	171	155
Fibrinogen	390	420	560	610	767	580	456	408	401	399	351	294	280	255
					Epinephrine					Epinephrine				
					5 days (4–5 times/day)					3 weeks (3 times/day)				

N.B. Reference Range: Lymphocytes: 1–4.8 × 10^9^/L, Ferritin: 30–400 ng/ml, D-dimer: 0–0.44 mg/L, CRP: 0–5 mg/L, LDH: 135–235 U/L, and Fibrinogen: 200–400 mg/dl

## Discussion

The CS represents the most furious and serious complication of SARS-CoV-2. It is due to an excessive immune response to the virus and abundant release of proinflammatory cytokines into the circulation [22]. CS score (CSs) was proposed to accurately identify patients who are in this hyperinflammatory state. CSs is considered positive if there is lymphopenia and at least two other inflammatory markers of either: serum levels of ferritin, D-dimer, CRP, or LDH, are elevated [23]. Lymphopenia is defined as a lymphocytic count below 1 × 10^9^/L as reported in severe cases [24–26]. Our patient showed lymphopenia (0.92 × 10^9^/L). Ferritin is marker of inflammation and is elevated in cases associated with CS. Mean value reported in severe cases [27–29] was 800 ng/ml, while in our case, it was 2000 ng/ml. D-dimer is a fibrin degradation product, and median value correlated with severity [30–32] is 1 mg/L, while in our case it was 1.48 mg/L. CRP levels are considered an independent risk factor for poor prognosis and at a cutoff level of 100 mg/L is associated with mechanical ventilation and mortality [33]. Our patient’s value was 164.8 mg/L. LDH is a marker of tissue damage that is related to severe cases [24] with a mean value > 300 U/L. Our case had a value of 317 U/L. Fibrinogen is a clotting factor that increased during inflammatory response and the cutoff level to predict ICU admission [34] was 571.0 mg/dl. In our case, it was 767 mg/dl.

**Fig. 1:**
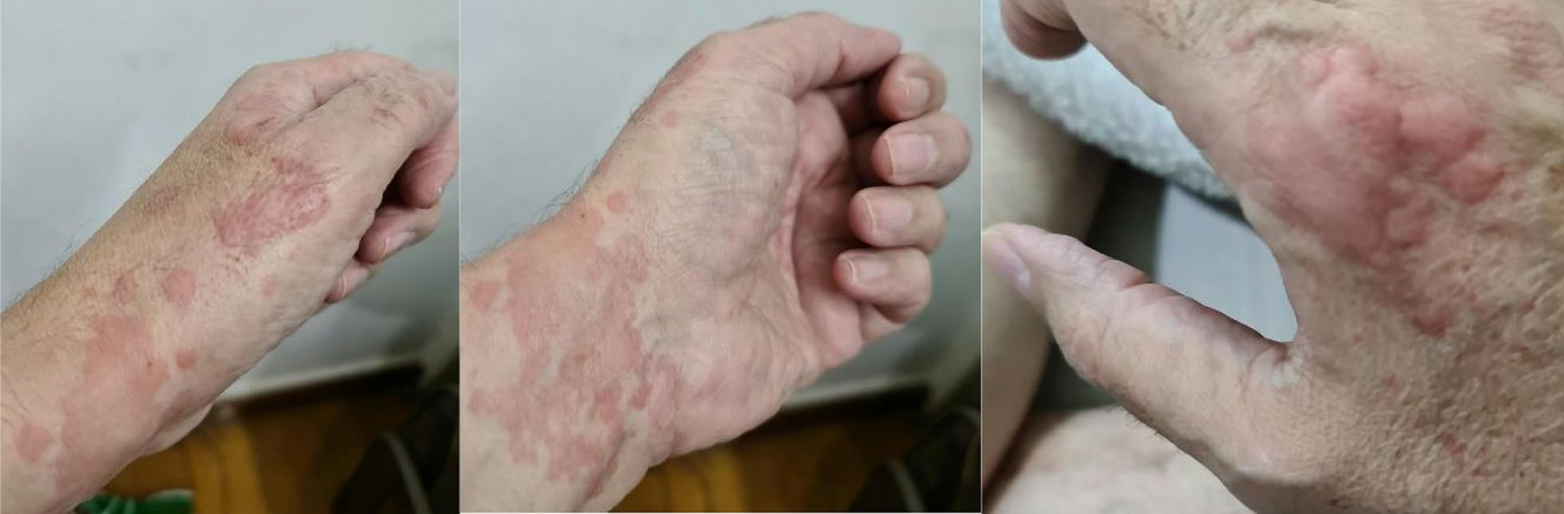
Part of the generalized skin rash

**Fig. 2:**
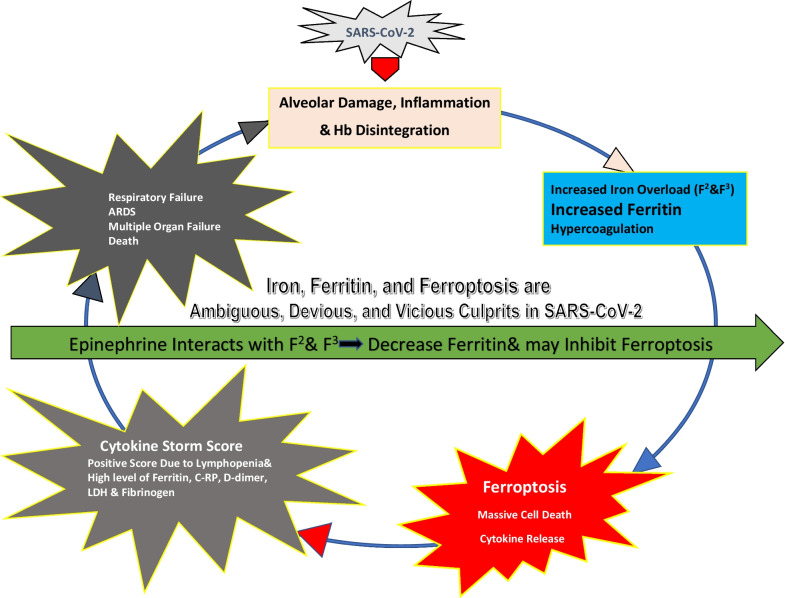
Epinephrine is cutting the vicious circuit of iron, ferritin, and ferroptosis which are the ambiguous, devious, and vicious culprits in SARS-CoV-2. Epinephrine interacts with F^2^ and F^3^ to decrease ferritin that may attenuate cytokine storm and inhibit ferroptosis. *ARDS* adult respiratory distress syndrome, *Hb* hemoglobin, *F*^*2*^ ferrous iron, *F*^*3*^ ferric iron, *CRP* C-reactive protein, *LDH* lactate dehydrogenase

Acute urticarial lesions have been noticed in several SARS-C0V-2 case series and typically are characterized by erythematous slightly raised papular rash followed by intense pruritic sensations [35]. Pruritus was reported in 92% of patients with urticarial lesions and was associated with a severe infection [36]. Herrero-Moyano et al. [37] proposed that a CS could be the cause of these rashes rather than the virus itself. Our case (Fig. 1) showed also an acute onset of urticarial lesion that coincided with the peak values of inflammatory markers. CS usually occurs in the second week of infection [38] which was the same time of urticarial rash, itching, tongue, and lips swellings as well as peak level of fever, ferritin, and other inflammatory markers in our patient. The time between mechanical ventilation due to respiratory failure and recognition of a positive CSs was found to be 12–96 h [23]. The timely control of CS through immunomodulators and cytokine antagonists is the key to reduce the mortality rate [39]. In our case, regular treatment and Epi may have successfully attenuated the CS as shown by decrease in ferritin and other inflammatory markers during the 5 days following the use of Epi (Fig. 2). This case report shows a clear-cut temporal association between Epi administration and rapid clinical and biochemical improvement in our patient with positive CSs. This may be explained by the interaction between Epi and labile plasma iron which may inhibit ferroptosis. The interaction between ferritin and Epi was first reported in 1956, and it was concluded that circulating ferritin can inhibit the vasoconstrictor response to Epi in an experimental study [40] (Table 2). More recently, it was found that EPI interacts with ferric (Fe^+3^) or ferrous (fe^+2^) iron from plasma labile iron pool and results in impeding activation of adrenergic receptors experimentally [41]. This explains lack of tachycardia and hypertension, which might be harmful to our patient.

**Table 2:** Epinephrin, from discovery of adrenal gland and isolation of the hormone, till synthetization as a drug and providing indications

Date	Event
1564	Bartolomeo Eustachio, anatomist, was the first to describe the adrenal gland [42]
1855	Thomas Addison described 10 cases with the clinical syndrome of adrenal insufficiency [43]
1894	Oliver and Schafer demonstrated the hormonal pressor effect of the adrenal extract, this was on Saturday, March 10, which was a day of note for medicine [44]
1899	Abel published a paper announcing an extract which he named “epinephrin” (the Greek word, epi, means “close by,” while nephros, “kidney”) [45]
1900	Takamine and Uenaka visited Parke, Davis & Co. for the full-scale production of adrenalin at the factory level. Coining the Name “Adrenalin” the Latin word “ad” means “near” while “renal” means “kidney,” and Patent Application [46]
1903	George Crile a surgeon who discovered the most important role for adrenaline in surgical shock [47] and cardiac arrest [48]
1903	Bullowa and Kaplan had described the successful treatment of asthmatics with subcutaneous injections of adrenaline [49]
1919	Harris Boughton reviewed a number of deaths in asthmatics with known allergies to horses and reported adrenaline use in anaphylaxis [50]
1923	Carl Bodon published a review of the use of intracardiac drugs including adrenaline [51]
1956	Green et al. reported the experimental finding that circulating ferritin inhibit the vasoconstrictor response to adrenaline [40]
2020	Jacic, Jelena Korac et al. Ferrous iron binding to epinephrine promotes the oxidation of iron and impedes activation of adrenergic receptors [41]
2023	Clinical report of reducing ferritin level by epinephrin which may attenuate cytokine storm by inhibiting ferroptosis

Surgical shock [47] and cardiac arrest [48] are the first clinical indications of Epi that were discovered by Crile in 1903 (Table 2). Epi may present a therapeutic option to lower increased iron stores, reflected by high serum ferritin levels, associated with type 2 diabetes mellitus (T2DM) [52] and other cardiometabolic diseases such as coronary heart disease (CHD) and cerebrovascular disease (CEVD) [53]. It is concluded that inhibiting ferroptosis significantly reduces ischemia/reperfusion-related cardiac injury [54] and may also suppress inflammation and improve wound healing in diabetic ulcer [55].

## Conclusions

Epi interacts with ferric and/or ferrous iron to build a stable complex that impedes activation of beta-adrenergic receptors. Epi may attenuate cytokine storm and decrease ferritin levels associated with viral and medical diseases such as T2DM and cardiometabolic diseases. Epi may inhibit ferroptosis thus significantly reduces ischemia/reperfusion-related cardiac injury and may improve wound healing in diabetic ulcer. As a new clinical indication of Epi, extensive studies are required for further assessment and possible therapeutic uses.

## Data Availability

All data related to this case report are contained within the manuscript.

## Abbreviations

SARS-CoV-2: Severe acute respiratory syndrome coronavirus 2
COVID-19: Coronavirus disease 2019
ARDS: Acute respiratory distress syndrome
CS: Cytokine storm
Epi: Epinephrine
T2DM: Type 2 diabetes mellitus
CMDs: Cardiometabolic diseases
CHD: Coronary heart disease
CEVD: Cerebrovascular disease
ICU: Intensive care unit
ROS: Reactive oxygen species
Fe^+2^: Ferrous iron
Fe^+3^: Ferric iron
CRP: C-reactive protein
LDH: Lactate dehydrogenase
